# Oral Chronic Graft-Versus-Host Disease

**DOI:** 10.3389/froh.2022.903154

**Published:** 2022-05-20

**Authors:** David Dean, Herve Sroussi

**Affiliations:** ^1^Department of Oral Medicine, University of Washington/Seattle Cancer Care Alliance, Seattle, WA, United States; ^2^Division of Oral Medicine and Dentistry, Brigham and Women's Hospital and Dana Farber Cancer Institute Department of Oral Medicine, Infection and Immunity, Harvard School of Dental Medicine, Boston, MA, United States

**Keywords:** chronic GVHD, hematopoietic cell transplantation, oral medicine, dental, supportive care

## Abstract

Chronic oral graft-versus-host disease (cGVHD) is a complex, frequent, and highly impactful complication of allogeneic hematopoietic cell transplantation (alloHCT). It represents the leading cause of morbidity and mortality in long-term alloHCT survivors. cGVHD can affect almost any visceral organ system and commonly affects the skin, eyes and mouth, manifesting with signs and symptoms similar to other known immune-mediated and autoimmune diseases. Oral manifestations of GVHD include inflammation, thinning, and ulceration of oral mucosal tissues (similar to lichen planus), lymphocyte-mediated salivary gland dysfunction (similar to Sjögren/Sicca Syndrome), and decreased oral opening (trismus) secondary to sclerosis of oral and perioral tissues (analogous to limitation in scleroderma). Potential sequelae include severe mucosal pain, compromised nutrition, weight loss, limitation in opening, and sometimes irreversible fibrosis of the salivary glands. While some cases can be managed with topical therapies, management may also require long-term targeted immunosuppressive and/or corticosteroid therapy with associated risk of local and systemic infection, hyperglycemia, kidney dysfunction, osteopenia/osteoporosis, and possibly secondary malignancies. The aim of this mini-review is to provide an up-to-date review of literature related to the diagnosis and management of oral cGVHD to aid dental and medical clinicians in optimizing oral cGVHD therapy while minimizing potential adverse effects.

## Introduction

Chronic graft-versus-host disease (cGVHD) is a common, pleotropic disorder with distinct manifestations throughout the body. cGVHD is diagnosed in 30–50% of allogeneic hematopoietic cell transplantation (alloHCT) recipients with more than 90% diagnosed within 12 months [1–3]. Incidence is increasing due to greater frequency of alloHCT, improved survivorship, and trends in donor selection, graft source, and other factors [2, 4]. It represents the leading cause of morbidity and mortality in long-term survivors otherwise in remission from their hematological disease [5–11].

## Oral cGVHD

Oral cGVHD is characterized by lichenoid mucositis, immune-mediate salivary dysfunction, and tissue sclerosis. Recent studies suggest that each represents a discrete clinical entity with little interrelationship [12, 13]. Though oral cGVHD is not independently associated with mortality, it may cause significant morbidity, making oral therapy an important component in comprehensive management [13–18].

**Figure 1:**
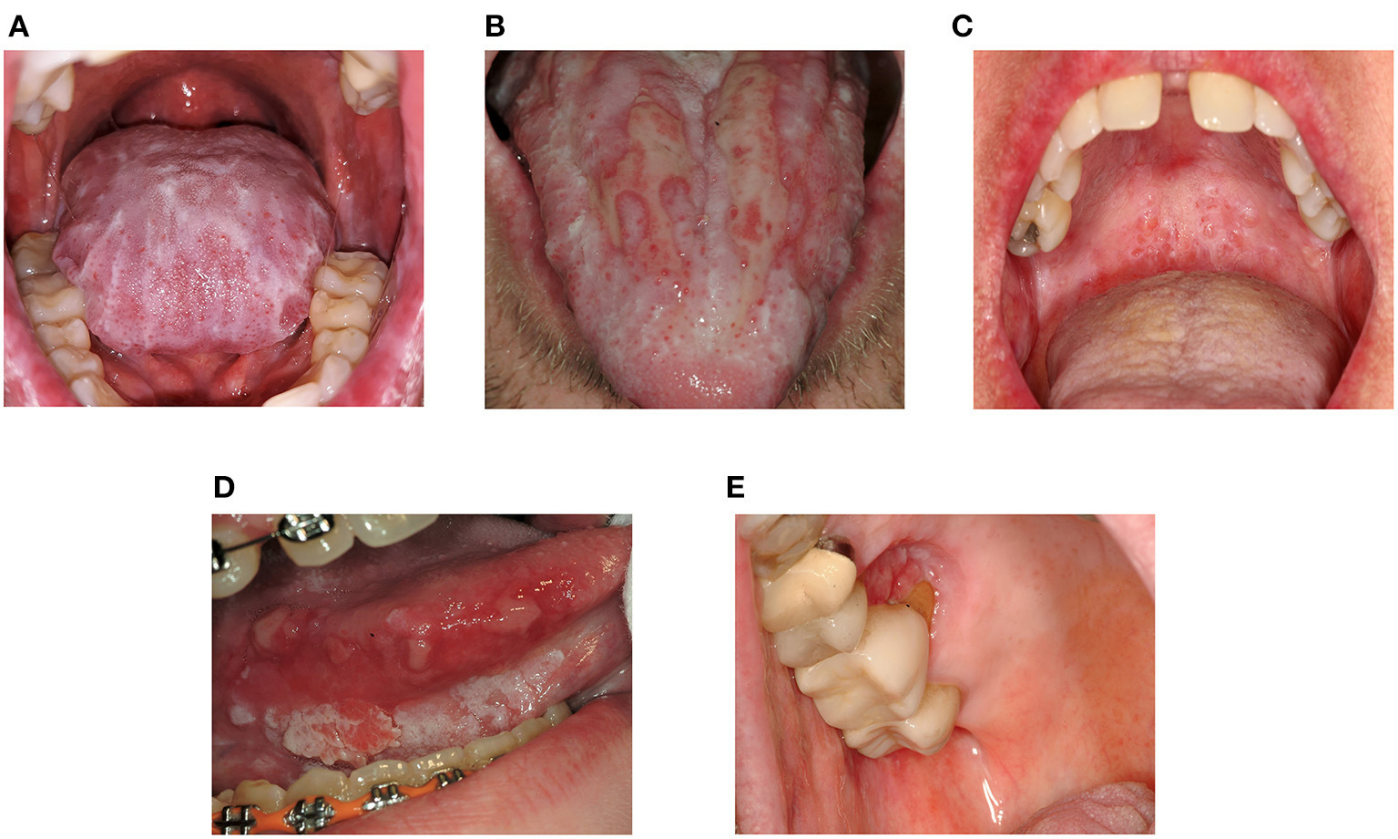
Clinical features of oral mucosal cGVHD and Oral Squamous Cell Carcinoma. **(A)** Dense lichenoid reticulations involving the dorsal tongue **(B)** Pseudomembrane-covered ulcerations of the dorsal tongue surrounded by lichen-like changes (lichenoid hyperkeratosis) **(C)** Superficial mucoceles of the left soft palate. Note the prominent minor salivary glands and thin lichenoid striations affecting the hard and soft palates **(D)** Squamous cell carcinoma of the right ventrolateral tongue in a patient with longstanding oral cGVHD **(E)** Squamous cell carcinoma of the right hard palate at a site of persistent cGVHD involvement.

Symptoms in mucosal cGVHD range from asymptomatic lichenoid changes to severely painful ulcerations which can be disabling (Figures 1A,B). Often minimal pain is reported at rest, though thinning and ulceration of the oral mucosa regularly cause sensitivity to previously tolerated stimuli [19, 20]. Common triggers include acidic, spicy or highly seasoned foods, carbonated beverages, alcohol/alcohol-based products, and flavoring agents such as mint in toothpaste [14, 19–22]. Tissue irritation may compromise nutrition, mastication, speech, swallowing, social interactions, and ability to perform effective oral hygiene, particularly when salivary dysfunction is also present [13, 23]. Mucosal ulceration compromises barrier function increasing risk for oral-sourced bacteremia [24].

Oral health and health-related quality of life are further impaired by immune-mediated salivary dysfunction which is especially impactful [12, 13]. Dysfunction is associated with xerostomia (the subjective impression of dry mouth), increased adherence of bacterial plaque and debris, difficulty swallowing, and decreased ability to clear viscous secretions. Qualitative and quantitative changes in saliva increase susceptibility to dental caries, oral candidiasis, and mucosal breakdown [25–28]. Dry mouth exacerbates mucosal symptoms while independently decreasing quality of life [13]. Mucoceles, dome-shaped fluid-filled “blisters” arising from minor salivary glands, are also common in cGVHD, though not specific to the disease [19, 22] (Figure 1C).

Function may also be limited by trismus resulting from oral sclerosis which has been described as a late effect of oral cGVHD. Pathophysiology is not well-understood [14, 20, 29]. Recent work found association between limited mouth opening and skin sclerosis, but not lichenoid mucositis suggesting a cutaneous rather than mucosal process [12]; however, limitation may be multifactorial as chronic inflammation can cause mucosal scarring [19, 22]. Trismus can impact nutrition, oral hygiene, and ability to comfortably complete dental procedures [22].

Taste-alterations have been described [30–32].

## Diagnosis and Staging of cGVHD

In 2014 the NIH Diagnosis and Staging Working Group revised standards established in 2005 [33] to clarify enrollment criteria for clinical trials, align disease staging with treatment prognosis, and aid in treatment selection [34]. Diagnostic criteria were updated for the skin, mouth, lungs, and genitalia and organ severity scores revised in eight organs to improve global severity scoring. Diagnostic and distinctive features are defined for each system with diagnosis confirmed by the presence of one diagnostic feature, or one distinctive feature supported by a confirmatory test (e.g., biopsy). Conversely, acute GVHD, which was initially defined by time of occurrence (<100 days post-alloHCT), is now diagnosed and staged based on rash, total bilirubin elevation, and diarrhea. Overlap of acute and chronic GVHD can be seen and may relate to worse clinical outcome [3]. Though diagnostic criteria are foundational, some patients with equivocal diagnoses may also require therapy to minimize adverse effects of alloimmunity [3].

### Diagnosis of Oral cGVHD

The mouth is commonly affected by cGVHD with up to 83% of cGVHD patients meeting diagnostic criteria [35, 36]. Oral cGVHD may co-occur with disease in other visceral organs or present as the initial or only site of involvement [14, 16, 22, 37]. The high incidence and ease of oral examination may aid in diagnosis of emerging disease [37]. Diagnosis is based on visual examination supplemented by history and global health status [14, 19]. Pathophysiology of oral cGVHD is not fully characterized, though findings of inflammation and fibrosis mirror other systems [3, 14]. Lichen planus-like changes are diagnostic and do not require confirmational biopsy. They may present as Wickham striae, lichenoid patches, or plaques. The 2014 NIH criteria replaced the term “hyperkeratotic plaques” [33] with “lichen-like changes” to differentiate from other causes of oral hyperkeratosis which may be reactive (frictional or chemical induced), infectious (pseudomembranous and hyperplastic candidiasis), or potentially malignant (idiopathic leukoplakia) [34]. Distinguishing cGVHD from idiopathic leukoplakia is especially important given increased risk of oral malignancy after alloHCT [38–43].

Distinctive features include mucosal atrophy, pseudomembranes, ulcers, mucoceles, and xerostomia. Distinctive features must be differentiated from other conditions common in the AlloHCT population including candidiasis, recurrent herpetic infection, drug reaction, mucosal trauma, recurrent or primary malignancy, and salivary dysfunction secondary to xerogenic medications and/or polypharmacy [19, 29, 34]. Biopsy, culture, viral PCR, and sialometry may be valuable in confirming diagnosis [32]. When cGVHD is suspected, biopsy should be obtained from non-ulcerated tissue and reviewed by an experienced pathologist [22, 44].

### Severity Scoring and Response Criteria

Severity scores are used to quantify organs affected by cGVHD and resulting level of functional impairment [34]. Global and organ-specific scores are used in treatment planning which is strongly influenced by extent and severity of disease [34, 45, 46]. Topical and local therapies are favored in mild cases (confined to ≤ 2 non-respiratory organs each with a maximum score of 1) [3, 45] while systemic therapy is often required in moderate to severe disease. The NIH Global Severity Score is a simple instrument that can be used by non-specialists to assess functional impact of cGVHD [34]. Eight organs are scored from 0 to 3 with higher scores indicating greater disability [34, 36]. An oral score of 1 reflects disease that is not significantly impacting nutritional intake while a 3 indicates major dietary limitations caused by oral symptoms [34]. Asymptomatic oral lichenoid changes do not impact global scoring as they do not affect nutrition [34]. Patients should also rate their peak sensitivity (i.e., irritation from normally tolerated stimuli) over the past week using a 0 to 10 scale either alone or in combination with the oral questions on the Lee cGVHD symptom scale [46, 47].

Organ-specific response criteria are intended for use by specialists to capture higher level detail [46]. The preferred oral instrument is the NIH Modified Oral Mucosa Score (OMRS) which assigns scores for mucosal erythema (0–3), lichen-like changes (0–3), and tissue ulceration [0–6] based on severity and surface area affected. Final scores range from 0 to 12 with scores of ≥2 representing clinically significant disease. Score change of ≥2 indicates disease progression (if increasing) or response to therapy (if decreasing) [46, 48, 49]. Mucoceles have been removed from the scale due to challenges in reporting and lack of correlation with clinical outcomes [18, 46, 50, 51]. Lichenoid changes, erythema, and symptoms scores are most strongly associated with perceived change in disease status [37].

## Prophylaxis and Management

GVHD prophylaxis and treatment are complex and determined by extent of disease, co-occurrence of acute and chronic GVHD, degree of functional impairment, and patient specific factors including likelihood of end organ damage [45, 46, 52]. Therapy must balance GVHD-associated morbidity against the benefit of the graft-versus-leukemia (GVL) effect which decreases risk for relapse [53–55]. Standard prophylaxis includes a calcineurin inhibitor and an antimetabolite (most commonly methotrexate) [45]. Systemic corticosteroids, specifically prednisone 1 mg/kg, is well-established as first-line therapy [3, 45], though mild localized disease may be limited to topical or local corticosteroids. Addition of a non-steroidal agent in initial therapy has not shown additional benefit in patients with standard risk GVHD [56–58]. Non-steroidal immunomodulatory medication should be added in in severe GVHD to limit end organ damage and adverse effects associated with extended systemic corticosteroid use [45, 59]. Second-line therapy varies widely. Choice is often center-specific as studies comparing relative effectiveness are lacking [45]. Sirolimus, tacrolimus, cyclosporine, mycophenolate mofetil, pentostatin, and extracorporeal photopheresis are commonly used off-label [52]. The FDA has only been recently approved ibrutinib (2017) [60], ruxolitinib (2021) [61] and belumosudil (2021) [62] in the treatment of cGVHD.

While systemic immunomodulating medications are essential in treatment, they are associated with a variety of adverse effects including impaired immune function, decreased bone density, diabetes, renal dysfunction, neurologic side effects, and in some cases secondary malignancy [32, 63]. Risk mitigation is critical as most patients require systemic therapy beyond 2 years with up to 15% extending past 7 years [14, 56, 64]. Optimizing non-systemic therapies may help to limit prolonged use, particularly when the oral cavity is the primary site of involvement [16, 23, 32, 65, 66].

### Oral Mucosal cGVHD

Topical therapies are the cornerstone of oral cGVHD management and are valuable even when systemic treatment is required as combined therapy has greater effect than systemic alone [32, 65–68] (Table 1). Furthermore, the mouth is one of few organs in which aggressive topical therapy may successfully manage moderate-to-severe disease [65]. Oral cGVHD should be treated when there is loss of barrier function and/or when oral sensitivity is negatively affecting quality of life [14, 20, 32, 67]. Asymptomatic lichenoid changes do not require therapy as treating to “disease resolution” has limited clinical benefit. This practice is consistent with treatment approaches in other organs [69]. Data from cGVHD Consortium sites has confirmed that treatment behavior follows these recommendations with topical therapies more likely to be used in patients reporting pain and decreased oral function [70]. Follow-up is recommended to confirm symptom control and restoration of mucosal integrity. Therapy should be tapered over time to the lowest frequency (and potency) required to maintain effectiveness in symptomatic mitigation [16, 23].

**Table 1:** Topical therapies for oral mucosal cGVHD.

**Topical Therapies**	**Standard Instructions:**
Rinses: • Budesonide 0.03%^*^ • Dexamethasone 0.01%^*^ • Clobetasol propionate 0.05%^*^ • Tacrolimus solution 0.1%^*^ • Cyclosporine 100 mg/ml^#^ • Azathioprine 5 mg/ml^#^	Rinse and hold 5 to 10 mL for 3 to 5 min and spit out. Repeat 1 to 4 times daily. Rinses are followed by a 20 to 30-min period of no food or fluid intake.
Ointments/gels • Tacrolimus ointment 0.1%^*^ • Clobetasol propionate gel 0.05%^*^ • Fluocinonide gel 0.05%^*^ • Azathioprine gel (5 mg/ml in methylcellulose base)^#^	Apply a small amount of ointment/gel (“pea size”) directly to the lesion (s) for 10 to 15 min. Gauze occlusion may help to hold in place. Trays for gingival application can be fabricated. Applications are followed by a 20 to 30-min period of no food or fluid intake.

* *Supported by prospective clinical trial*.

# *Supported by case series or retrospective studies*.

Medication selection is based on the extent of oral lesions, medication potency, cost, availability, and patient preference [19, 23]. There are currently no FDA-approved topical therapies for oral cGVHD, though corticosteroids and calcineurin-inhibitors are regularly used in practice. Rinses are recommended when lesions are widespread to facilitate application to all sites. Localized lesions may be treated with higher potency gels or ointments which can be applied under gauze occlusion to maximize local effect. Numerous topical steroids, topical non-steroidal agents (e.g., tacrolimus [71–75], cyclosporine [76], sirolimus [77], azathioprine [78, 79], thalidomide [80]), and phototherapies (Photobiomodulation [81], PUVA [82, 83], UVB [84]) have been used in clinical practice and excellent evidence summaries have been previously published in national and international consensus documents [32, 65, 85, 86] and comprehensive reviews [14, 22, 66]. Surveys indicates that over 90% of specialists initially favor topical steroids with tacrolimus the preferred second-line alternative [87]. Among topical therapies only clobetasol, dexamethasone, tacrolimus solution and budesonide effervescence tablets have been analyzed in randomized trials [68, 72, 88]. Evidence-based practice is hindered by availability as only dexamethasone and prednisolone solutions are commercially manufactured in the United States [23, 65]. Other agents may be compounded; however, this increases cost to the patient as compounded medications are unlikely to be covered by insurance [23].

Topical steroids are generally well-tolerated, but patients must be monitored for potential adverse effects. Secondary candidiasis with topical steroids is not uncommon and [63, 88, 89] and prophylactic antifungal coverage is often used in clinical practice [16, 65, 87]. Risk factors for oral candidiasis, including immunosuppression, altered quantity and composition of saliva, and the use of medications that alter the normal oral flora (e.g., antibiotics, steroid inhalers), are common in people living with cGVHD. Candidiasis should therefore be considered when presumed oral cGVHD is not responsive to topical steroids. Whereas systemic uptake of tacrolimus has been described in case series and blood levels should be periodically monitored to rule it out [75]. Limited data is available for topical steroid absorption in the cGVHD population. Nonetheless patients should be monitored for cushingoid features or other signs of adrenal suppression [22]. The best available evidence is a study of 62 patients with severe erosive lichen planus treated with clobetasol propionate 0.05% rinse (10 mL for 5 min TID for 2 to 6 weeks based on response). Plasma cortisol levels showed signs of suppression in 85.5% of patients during initial therapy with only 4% of patients effected in the maintenance phase (suggesting lower systemic absorption after mucosal integrity is reestablished [90]. There were no major adverse events and dose reduction was effective in reversing cushingoid features and capillary fragility. Topical budesonide has been proposed as a preferred alternative for extended use due to low transmucosal absorption and poor systemic bioavailability [68].

Topical therapies may also be useful adjuncts in lesion assessment, though biopsy is required for definitive diagnosis. For example, short (e.g., 2 week) therapeutic trials of high potency topical steroids have been recommended in differentiation of lichenoid hyperkeratosis from leukoplakia. Immune-mediated lesions, such as cGVHD, are likely to respond to topical therapy while oral potentially malignant lesions will not. Non-responsive lesions should be biopsied to rule out epithelial dysplasia and/or squamous cell carcinoma [32, 65]. Intralesional injections with triamcinolone acetonide (40 mg/mL) have also shown value in treatment of persistent oral ulcerations which must also be differentiated from oral malignancy [91].

### Salivary Dysfunction

Treatment of salivary dysfunction focuses on reestablishing oral lubrication to improve comfort and function while simultaneously minimizing risk of dental sequelae related to hyposalivation. Patients are encouraged to take frequent sips of water to moisten the mouth and maintain hydration. Liquid intake during mealtime, or when swallowing medications, can help to limit dysphagia if swallowing function is otherwise normal. Sugar-free candy, mints, and chewing gum can provide gustatory and masticatory stimulation to glands to increase salivary flow during the day [92]. All are inexpensive and widely available without a prescription. Normal saline rinses, over-the-counter coating agents, in the form of rinses, sprays, and gels, and artificial saliva may temporarily reduce xerostomia, but must be regularly reapplied [16, 32, 65]. Adhesive tablets, such as XyliMelts^®^, slowly dissolve over time while simultaneously stimulating flow. They may be especially useful during sleep along with other longer lasting agents (e.g., gels, oil-based products).

Systemic sialagogues are commonly used off-label in the treatment of severe cGVHD-related salivary dysfunction. Pilocarpine is FDA-approved for the treatment of radiation-induced dry mouth in head and neck cancer patients and the treatment of dry mouth and dry eyes in Sjögren syndrome, while Cevimeline is approved in Sjögren syndrome only. Data in the cGVHD population is limited [93, 94]. Daily use of pilocarpine has been associated with increased salivary output, improved oral function, and restoration of normal sialometric properties, though data is limited to one randomized trial and several open enrollment studies [95–98]. Cevimeline has shown similar safety and efficacy in other populations [99, 100], and case series suggest it is an effective alternative in cGVHD [101]. A survey of practitioners in specialty health centers confirmed pilocarpine as the most common first-line therapy for salivary dysfunction in cGVHD (41.7%) with saliva substitutes favored as first-line palliative therapy [102]. The FDA cites hypersensitivity, uncontrolled asthma, acute iritis, and narrow angle glaucoma as contraindications [103] while consensus guidelines in cGVHD also advise against use in patients with cardiac disease and obstructive pulmonary disease (including pulmonary GVHD) [65]. Preexisting pulmonary and gastrointestinal GVHD may be exacerbated due to increase in bronchial and gastric secretions [16, 19, 87]. Sweating and flushing, commonly reported in other populations, is reported to be uncommon in the cGVHD population [19]. Titration of the medication over 2 weeks can be helpful in mitigating this effect when present. Side effect profiles are similar, and choice may be based on relative out of pocket cost [19].

### Oral Sclerosis

Stretching and physical therapy are the most common therapies for oral and perioral sclerosis. Use of long-term, sequential intralesional steroid injections have also been described [65].

### Palliative and Ancillary Therapy

During active therapy, palliative and ancillary therapies can be helpful to decrease pain, mucosal trauma, secondary infection, and dental sequelae. Dietary modifications, favoring bland and soft foods, and adjustments to oral hygiene practices (e.g., soft bristle toothbrush, non-mint and sodium lauryl sulfate-free toothpaste, non-alcohol-based rinses) can help to decrease mucosal sensitivity [19, 20]. Topical adhesive agents, such as ZilactinB^®^ or Orabase^®^, can be applied to localized ulcerations to decrease pain and recurrent trauma while occlusal guards or Essix retainers can minimize frictional irritation from sharp or malpositioned teeth. Effective plaque control and judicious use of topical antimicrobials, such as chlorhexidine gluconate, can decrease gingivitis improving gingival comfort and decreasing risk for secondary infection [20].

Topical anesthetics, such as lidocaine or benzocaine, are recommended if soft tissue pain is limiting ability to eat or perform effective oral hygiene. Numerous preparations are available, though 2% viscous lidocaine is favored in consensus documents [32, 65, 67]. Care should be taken to avoid trauma after application, particularly in pediatric patients [65]. Gargling and swallowing should be avoided to decrease risk for aspiration [65]. Studies in oral mucositis patients have shown low systemic absorption and risk is low with oral application when used as directed [104]. Methemoglobinemia has been described after application to large mucosal surfaces (e.g., bronchoscopy) and with excessive oral use [105–108].

## Late Oral Complications

### Dental Caries

Patients with salivary cGVHD are at high risk for dental caries. Extensive caries has been described within 2 years of diagnosis [27]. Prevention is paramount as progressive caries increases risk for oral-source infection, the need for invasive dental procedures, and overall cost of care. Dietary counseling is recommended to limit refined sugar and other fermentable carbohydrates [109, 110]. Daily application of prescription fluoride gel is recommended and may be supplemented by professional fluoride application and shorter recall intervals in cases of severe hyposalivation [20, 32, 65, 87].

### Second Malignancy

NIH consensus recommendations call for annual oral examinations in AlloHCT patients surviving beyond 1 year [111] due to increased risk for oral cancer [39–43, 112] (Figures 1D,E). Elevated risk for oral squamous cell carcinoma in oral cGVHD patients has prompted other groups to recommend more frequent assessment [87] analogous to other oral premalignant disorders [113]. Long-term follow-up is essential as the risk increases over time [114–117].

## Summary

Oral cGVHD is a common complication of AlloHCT associated with decreased quality of life. Oral cGVHD includes three distinct manifestations: lichenoid mucositis, salivary gland dysfunction, and tissue sclerosis resulting in trismus. Complications include tissue sensitivity, loss of mucosal integrity, infection risk, xerostomia, and compromised oral function. Topical, intralesional, palliative, and ancillary interventions are essential in managing these complications and may help to limit prolonged use of systemic immunomodulatory agents. Long-term follow-up is essential due to elevated risk for oral cancer which increases with time.

## Author Contributions

DD drafted and critically revised the manuscript. HS critically revised the manuscript. All authors give final approval and agree to be accountable for all aspects of the work.

## Funding

Research reported in this publication was supported by the National Institute of Dental and Craniofacial Research of the National Institutes of Health under award number R01DE028336 which funded the entirety of the work. The content is solely the responsibility of the authors and does not necessarily represent the official views of the National Institutes of Health.

## Conflict of Interest

The authors declare that the research was conducted in the absence of any commercial or financial relationships that could be construed as a potential conflict of interest.

## Publisher's Note

All claims expressed in this article are solely those of the authors and do not necessarily represent those of their affiliated organizations, or those of the publisher, the editors and the reviewers. Any product that may be evaluated in this article, or claim that may be made by its manufacturer, is not guaranteed or endorsed by the publisher.
